# Parsplana vitrectomy alone versus parsplana vitrectomy combined with phacoemulsification for the treatment of rhegmatogenous retinal detachment: a randomized study

**DOI:** 10.1186/s12886-021-01954-y

**Published:** 2021-05-03

**Authors:** Paolo Mora, Stefania Favilla, Giacomo Calzetti, Giulia Berselli, Lucia Benatti, Arturo Carta, Stefano Gandolfi, Salvatore A. Tedesco

**Affiliations:** 1 Ophthalmology Unit, Department of Medicine and Surgery, University Hospital of Parma, via Gramsci 14, 43126 Parma, Italy; 2 Independent Researcher, on behalf of the University of Parma, Parma, Italy; 3 Institute of Molecular and Clinical Ophthalmology Basel, Basel, Switzerland

**Keywords:** Rhegmatogenous retinal detachment, Parsplana vitrectomy, Phacovitrectomy, Retinal detachment recurrence, Cataract

## Abstract

**Background:**

To compare parsplana vitrectomy (PPV) with and without phacoemulsification to treat rhegmatogenous retinal detachment (RRD).

**Methods:**

Subjects aged 48–65 years with RRD in a phakic eye due to superior retinal tears with an overall extension of retinal breaks < 90° underwent to PPV alone (group A); or PPV plus phacoemulsification (phacovitrectomy, PCV, group B). Post-operative follow-up visits occurred at 1 week, 1 month (m1), 3 months (m3), and 6 months (m6) after surgery. The main outcome was the rate of retinal reattachment. Secondary outcomes included best-corrected visual acuity (BCVA), intraocular pressure (IOP), central macular thickness (CMT), and cataract progression (in the lens-sparing [PPV-alone] group).

**Results:**

In this initial phase of the study a total of 59 patients (mean age: 55 years, 59 eyes) were enrolled: 29 eyes in group A and 30 eyes in group B. Both groups had similar gas tamponade. During the follow-up there were three cases of RRD recurrence in group A and one in group B. The relative risk of recurrence in group A was 3.22 times higher but the difference was not significant (*p* = 0.3). The two groups were also similar in terms of BCVA and IOP variation. At m3, CMT was significantly higher in group B (*p* = 0.014). In group A, cataract progression was significant at m6 (*p* = 0.003).

**Conclusions:**

In a cohort of RRD patients selected according to their preoperative clinical characteristics, PPV was comparable to PCV in terms of the rate of retinal reattachment after 6 months.

**Trial registration:**

ISRCTN15940019. Date registered: 15/01/2021 (retrospectively registered).

## Background

Rhegmatogenous retinal detachment (RRD) is a sight-threatening disease started by the formation of a retinal break. When the separation of the neurosensory retina from the retinal pigment epithelium has occurred, surgical management is required. The incidence of RRD has been reported to be between 6.3 and 17.9 per 100,000 population [1]. RRD is largely more common in adults with myopia or pseudophakic eyes but it can affect younger patients, particularly those with risk factors such as ocular trauma or hereditary ocular abnormalities [2–4]. Although scleral buckling surgery still plays a role in the management of some types of RRD, in recent years there has been a trend toward parsplana vitrectomy (PPV) as the primary procedure, maybe also because young surgeons are particularly familiar with this latter technique [5]. For phakic RRD, PPV can be performed with or without removing the lens, and previous studies have reported advantages and drawbacks for both approaches [6–10]. Phacovitrectomy (PCV), i.e., phacoemulsification combined with PPV, is associated with: a) enhanced retinal visualization during posterior segment surgery; b) better access to the vitreous base allowing for a more extensive vitrectomy and endolaser treatment (thereby ensuring more extensive gas filling and better tamponade of retinal breaks; and c) reduced risk through avoiding the need for a second surgery, which can be technically challenging due of the lack of vitreous support. However, there are also disadvantages of PCV: a) there is a risk of postoperative refractive errors, especially in macula-off cases; b) iatrogenic anisometropia is frequent in myopic subjects; and c) it can lead to the removal of a largely healthy organ in cases of no/mild cataract with residual accommodative function. These latter issues can be avoided by performing a lens-sparing procedure (i.e. PPV-only).

PPV and PCV have been compared for the treatment of RRD in several recent large, retrospective studies [10–14]. One prospective series included two groups that underwent vitreoretinal surgeries for various pathologies [15]. Concerning the postoperative complications described in both techniques, the most dangerous for the visual prognosis include, besides the risk of endophthalmitis, the poor control of the intraocular pression (with either secondary glaucoma or hypotony), hemorrhage in the anterior and/or posterior segment of the eye, edema or morphological changes in the macular region, and the development of proliferative vitreoretinopathy (PVR) [11].

In this report, we present the results of a prospective randomized trial comparing PPV and PCV to treat RRD, with regard to the rate of retinal reattachment maintained during a 6-month follow-up (main outcome). As secondary outcomes, changes in best-corrected visual acuity, intraocular pressure, central macular thickness, and the progression of the cataract (for the PPV alone group) were also evaluated. To date, this is the largest randomized study comparing PPV alone and PCV to treat specifically the RRD.

## Methods

### Patients and study design

This hospital-based, parallel randomized study compared PPV with and without phacoemulsification of the lens for the treatment of RRD. This study, which was approved by the local Ethics Committee (registration number 569/2018) and adhered to the tenets of the Declaration of Helsinki and CONSORT guidelines, was conducted in the Ophthalmology Unit of the University Hospital of Parma (Parma, Italy) between December 2018 and November 2019. The inclusion criteria were as follows: 1) aged 48–65 years with RRD in phakic eyes; 2) the presence of up to three separate retinal tears within the superior 180° of the retinal circumference with an overall extension of retinal breaks < 90°; 3) PVR not exceeding the grade B according to the updated classification of 1991 [16]; and 4) lens opacity not exceeding the first grade of each category of the Lens Opacities Classification System III (LOCS III) scale [17]. The exclusion criteria were previous surgery in the affected eye (excluding corneal refractive procedures), current use of topical hypotensive medications, documented diabetic or hypertensive retinopathy, age-related maculopathy, and optic nerve vascular pathologies. The trial used equal randomization (i.e., 1:1) and the subjects were sequentially assigned to one of the two treatment groups by date of enrollment.

Patients scheduled for vitreoretinal surgery to treat RRD provided general and ocular medical history data, including current refractive correction, and underwent a slit lamp examination with applanation tonometry, lens opacity grading, and fundoscopy under mydriasis. Written informed consent was obtained from patients fulfilling the study criteria. All affected eyes of enrolled patients underwent preoperative optical coherence tomography (OCT; Cirrus HD-OCT 4000; Carl Zeiss Meditec, Dublin, CA, USA). In cases with macula-on RRD, the axial length (AL) was evaluated using optical biometry (IOL-Master; Carl Zeiss Meditec). In cases with macula-off RRD, AL was measured using the combined vector-A/B-scan ultrasound biometry (B-scan Plus; Accutome, Malvern, PA, USA) technique [18]. The optimal intraocular lens (IOL) power was calculated using the Sanders–Retzlaff–Kraff/theoretical (SRK/T) formula, for all eyes except those with prior refractive surgery (for which we used the Barrett Universal II formula). All surgeries were performed by the same two surgeons (PM/ST) under general anesthesia or local anesthesia with sedation, according to the patient’s general condition and predisposition. All patients were hospitalized on the day of surgery and discharged the next day after slit lamp examination of the anterior segment, fundus evaluation, and tonometry of the operated eye.

#### Group A: Lens-sparing technique (PPV-only)

PPV was performed using a binocular indirect ophthalmomicroscope (BIOM; Oculus, Wetzlar, Germany) for noncontact, wide-angle surgery. A 25-gauge trocar with a valved cannula (Alcon Laboratories Inc., Fort Worth, TX, USA) was inserted transconjunctivally in the inferotemporal, superotemporal, and superonasal quadrants, 4 mm posterior to the limbus. A 27-gauge twin bullet lights system was inserted at around the 1 and 11 o’clock meridians. This allowed the surgeon to independently indent the periphery externally with one hand and perform peripheral vitreous shaving with the other. All surgeries were performed using the Constellation Vitrectomy System (Alcon Laboratories Inc.). During the procedure, core vitrectomy was initially performed. When the posterior hyaloid was attached to the posterior pole, detachment was performed using the vitreous probe at an aspiration rate of 400 mmHg, without cutting. In all cases, visualization of the posterior hyaloid was facilitated by 0.05 mL of preservative-free triamcinolone acetonide (Taioftal®; Sooft Italia, SpA, Montegiorgio, Italy). Perfluorocarbon liquid (PFCL) was injected intravitreally up to around 2 disc diameters from the posterior edge of the less peripheral tear, to promote internal subretinal fluid drainage during fluid-air exchange. Cryopexy was used to freeze areas around the retinal breaks and those adjacent to the sclerotomies. Fluid–air exchange was then performed to remove all balanced saline solution and PFCL before gas tamponade (C3F8 at 18%). At the end of surgery, all trocars were removed and sutures were placed according to the watertight nature of the holes. A subconjunctival injection of 0.2 mL of gentamicin and dexamethasone solution was administered before the lid speculum was removed.

#### Group B: Phacovitrectomy

Standard phacoemulsification was performed systematically before the PPV through a 2.2-mm clear corneal incision, with implantation of a hydrophobic, acrylic, foldable monofocal IOL (TECNIS®; Johnson & Johnson, New Brunswick, NJ, USA). The subsequent vitrectomy procedure was the same as that mentioned above, except that argon endolaser treatment of the breaks was used instead of cryopexy.

#### Postoperative regimen

Both groups received from day 1 up to day 7 after surgery topical tropicamide 1% twice a day and a combination of tobramycin 0.3% and dexamethasone 0.1%, 6 times per day. From week 2 to week 5 after surgery the topical combination of tobramycin 0.3% and dexamethasone 0.1% was tapered to 4 times per day.

The possible requirement for ocular hypotensive therapy and/or local adjunctive steroids was considered on a case-by-case basis according to the findings described below.

#### Outcomes and follow up

Primary study outcome: the anatomical success rate, defined as retinal reattachment 6 months after primary surgery without reoperation (postoperative argon laser treatment on areas considered to be at risk for new rhegmatogenous events was not considered as reoperation).

Secondary outcomes: final best-corrected visual acuity (BCVA), intraocular pressure (IOP), central macular thickness (CMT) and progression of the cataract (group A only). For the statistical analysis, this latter parameter was expressed as the sum of the scores for the affected eye on every LOCS III scale category at the follow-up visits.

Follow-up: all parameters defining the primary and secondary outcomes were assessed during postoperative follow-up visits scheduled at 1 week (w1), 1 month (m1), 3 months (m3), and 6 months (m6) after surgery (± 7 days starting from m1). For BCVA, patients were asked to read the Early Treatment Diabetic Retinopathy chart, placed at 4 m, until no more letters were identified correctly. The letters properly identified were then counted, and the corresponding logMAR was recorded. For IOP, applanation tonometry was performed before slit lamp examination. Two readings were performed. In the case of a > 2 mmHg difference between the readings, a third one was obtained and the two closest values were averaged. For lens transparency, the state of the lens was assessed in full mydriasis with a slit lamp, by referring to the most similar grading photograph observed on a portable retro-illuminated chart showing the LOCS III classification system standard images.

Patients with retinal detachment recurrence dropped out of the study and were managed according to the most suitable procedure. Adverse events (related or unrelated to the study) occurring between study visits were recorded as having occurred at the time of the closest scheduled visit. An IOP value ≥25 mmHg was taken to indicate the use of hypotensive drugs. OCT evidence of cysts or diffuse edema in the macular region necessitated subtenon injection of triamcinolone acetonide (Taioftal®; Sooft Italia), unless clinically contraindicated [19]. Laser treatment was performed postoperatively if new tears occurred and postoperative examination showed an insufficient effect of primary laser treatment or cryopexy.

#### Sample size and statistical analysis

A difference up to 10% in the achieving of the primary outcome was assumed as non-inferiority margin between the two procedures (i.e. δ = 10%). By accepting a type I error of 5% and a type II error of 20% (i.e. a study power of 80%), a total sample size of 50 units (2 groups of 25 units) was required. The units correspond to the eyes assigned to each treatment group. The software used for the calculations is G * Power 3.1.9 [20].

Demographics and ocular baseline findings are given as percentages for categorical data and as mean ± standard deviation (SD) for continuous variables. All analyses were performed using R software [21]. The primary outcome (retinal reattachment) was evaluated using Kaplan-Meier analysis and the log-rank test; the relative risk (RR) of detachment was determined for groups A and B. The baseline group comparison for normally distributed variables was performed using Student’s t-test, and proportions were compared using Fisher’s exact test. All between- and within-group follow-up analyses were based on repeated-measures ANOVA (Analysis of variance) models. For post hoc contrast analysis, Fisher’s least significant difference test (95% family-wise confidence level) was applied (Table 2). For all variables, *p*-values < 0.05 were considered significant.

## Results

Fifty-nine eyes of 59 patients (36 men and 23 women, mean age: 55 years) were enrolled for the study and randomly allocated to group A (29 eyes) or group B (30 eyes). Demographics and preoperative ocular findings are provided in Table 1. At baseline, the two groups showed no statistical difference.

**Table 1:** Demographics and preoperative ocular findings

PREOPERATIVE	Group A	Group B	***p-***value
**SEX (% females)**	34%	43%	0.415
**AGE (years)**	54 ± 5	56 ± 7	0.255
**SE (Dioptres)**	−4.27 ± 4.90	− 3.18 ± 3.83	0.349
**IOP (mmHg)**	12.9 ± 3.5	14.1 ± 2.8	0.150
**MACULA ON-OFF (% ON)**	41.38%	33.33%	0.406
**LENS OPACITY (LOCS III)**	0.38 ± 0.77	0.5 ± 0.78	0.553

*SE* Spheric equivalent, *IOP* Intraocular pressure, *LOCS III* Lens Opacities Classification System III scale

Preoperatively, the presence of an incomplete posterior vitreous detachment (PVD) was observable in 8 eyes; of these 5 had PPV and 3 PCV. All surgeries were uneventful in terms of complications related to the lens (no capsule ruptures, crystalline lens contact, or IOL displacement), endophthalmitis, and procedures on the posterior segment. At the 1-day postoperative visit, the retina was attached in all operated eyes, although there were some cases of corneal edema and mild hyphema.

**Fig. 1:**
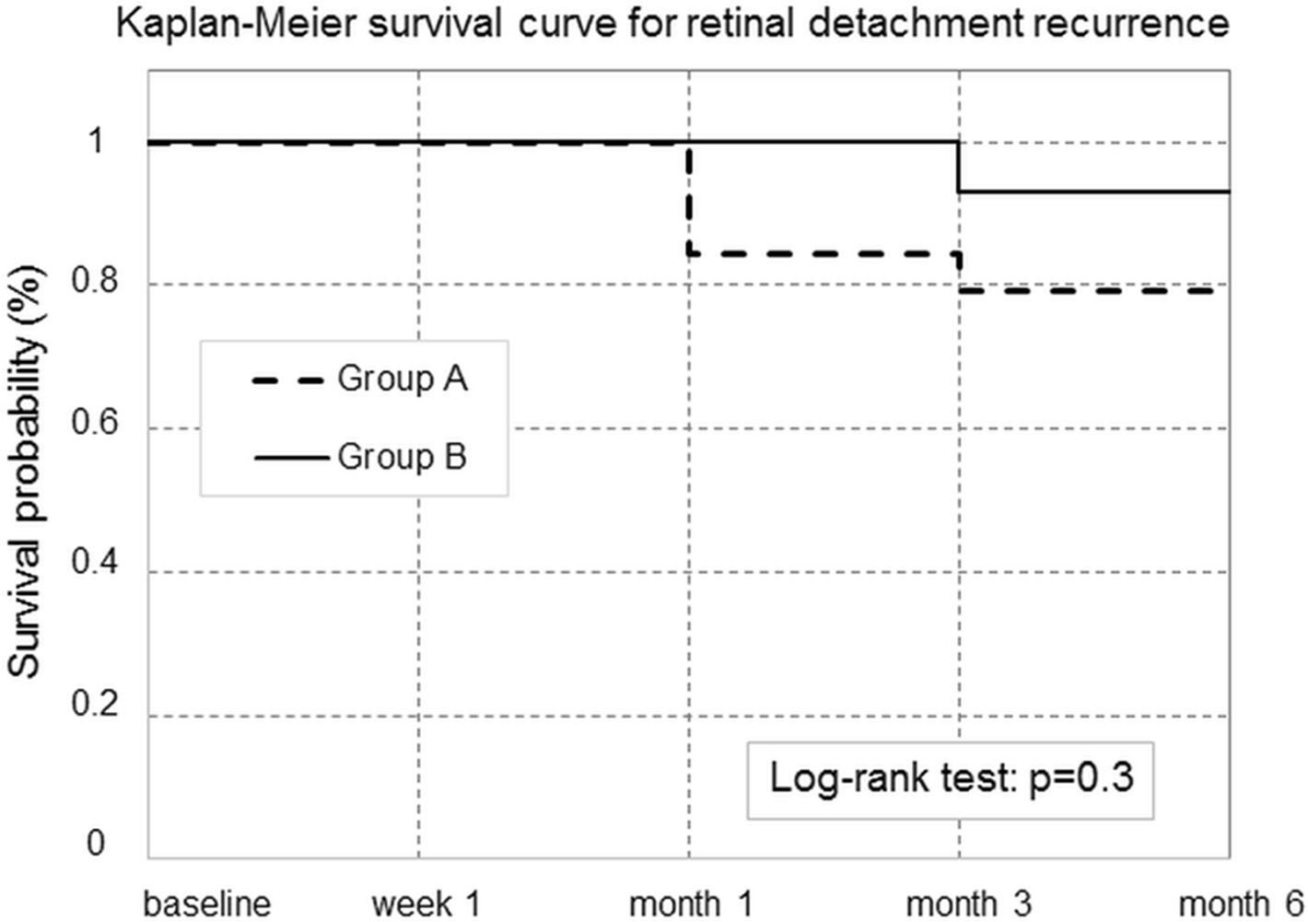
Kaplan-Meier survival curve for retinal detachment recurrence

RRD recurred in three cases in group A (two after 1 month and one after 3 months) and in one case in group B (at the m3 visit). The log-rank test revealed that the RR of RRD recurrence was 3.22 times higher in group A than in group B, but the difference was not significant (*p* = 0.3; Fig. 1).

Regarding the secondary outcomes, both within- and between-group (Table 2) analyses were performed. In both groups, BCVA improved over time and was thus significantly better at m6 than at w1 (*p* = 0.009 and *p* = 0.020 in groups A and B, respectively). The final mean BCVA in group A (0.19 ± 0.18 logMAR, Snellen equivalent of 20/32) was better than in group B (0.28 ± 0.23 logMAR, Snellen equivalent of 20/40), but not significantly (*p* = 0.203). The two groups differed in terms of the increase in BCVA over time (*p* = 0.016). In group A, the improvement was significant between w1 and m1 (*p* = 0.046); other changes throughout the subsequent follow up did not reach statistical significance. In group B, gradual increase in the BCVA was seen during the follow-up, but only the difference between w1 and m6 tested significant (*p* <  0.001).

**Table 2:** Evaluation of the secondary outcomes by between-group analyses

Secondary outcomes	Group	Time-points			
		Week 1 mean ± s.d.	Month 1 mean ± s.d.	Month 3 mean ± s.d.	Month 6 mean ± s.d.
**BVCA (logMAR)**	**A**	0.50 ± 0.51	0.26 ± 0.16	0.20 ± 0.21	0.19 ± 0.18
	**B**	0.55 ± 0.32	0.37 ± 0.26	0.31 ± 0.24	0.28 ± 0.23
	***p-*value**	n.s.	n.s.	n.s.	n.s.
**IOP (mmHg)**	**A**	17.7 ± 7.0	16.1 ± 2.4	15.9 ± 3.0	15.5 ± 2.6
	**B**	13.7 ± 3.3	15.4 ± 4.9	14.9 ± 3.3	14.2 ± 3.5
	***p-*value**	< 0.001	n.s.	n.s.	n.s.
**CMT (μm)**	**A**	–	279 ± 26	270 ± 26	270 ± 25
	**B**	–	278 ± 30	316 ± 118	297 ± 91
	***p-*value**	–	n.s.	0.01	n.s.
**LOCS III**	**A**	0.35 ± 0.75	0.54 ± 0.90	0.73 ± 1.15	1.31 ± 1.74
	***p-*value** **vs baseline**	n.s	n.s	n.s	0.003

*BCVA* Best-corrected visual acuity, *IOP* Intraocular pressure, *CMT* Central macular thickness (by Optical Coherence Tomography), *LOCS III* Lens Opacities Classification System III scale

Regarding the postoperative IOP values, a significant group difference was detected only at w1 higher in group A (*p* <  0.001). In group A, up to m3 the postoperative IOP was significantly higher than that preoperatively. Starting from m3, hypotensive treatment was prescribed in three eyes in group A, while it was administered in two eyes in group B starting from m1. In all cases, satisfactory IOP control was achieved.

The CMT, assessed based on the central subfield thickness as revealed by a 512 × 128 macular cube OCT scan (Cirrus HD-OCT 4000; Carl Zeiss Meditec), could be effectively analyzed only from m1 because of the poor quality of several of the earlier images (due to corneal edema and/or intraocular gas). The CMT was significantly greater in group B than group A at m3 (*p* = 0.014), with the two groups showing similar values thereafter. It should be noted that subtenon steroid injections were performed in five eyes of group B with cystoid macular edema at m3 (vs. 0 eyes in group A; *p* = 0.026 with χ^2^-test). Due to the presence of cystoid macular edema at m6, steroid injections were performed in four eyes in group B (for the second time in three eyes), whereas no injections were required in group A (*p* = 0.049 with χ^2^-test).

Finally, compared to the preoperative examination, the mean total LOCS III score in group A was significantly different from baseline only at the end of the follow up (i.e., at m6; 1.31 ± 1.74 vs 0.35 ± 0.75 at baseline, *p* = 0.003).

## Discussion

In our series, the rate of retinal reattachment at 6 months after PPV without cataract extraction (group A) was 89.7%; in the PCV group (group B), the rate was 96.7%. These data are consistent with those reported from prior studies dealing with the topic [13, 14, 22–24]. Group A had a RR of RRD recurrence 3.22 times higher than group B, but the difference was not significant. In total, we recorded four cases of recurrence, three of which were from previous macula-on RRD. All these first surgery failures were related to the occurrence of new retinal breaks in areas quite far from the original involvement. During the new surgeries, no clinically significant PVR was observed.

We think that in some RRD cases it is unsuitable to plan PPV alone in phakic eyes, particularly when: there is a large detachment with a traumatic etiology; extensive vitreous shaving is required (up to the base); there are tears suggestive of medium- to long-term viscous tamponade; or the lens is already showing opacity. Against this background, in our comparative study we included only cases of RRD in which lens-sparing surgery would be justified. The age selection minimized the likelihood of advanced cataract and absolute presbyopia; the other inclusion requirements were aimed to avoid some pre-existing characteristics which correlate with lower single operation success rate [25]. Interestingly, Caiado et al. reported better success rates for PCV or PPV in already pseudophakic eyes [12]. The higher RR in group A is in accordance with these results, and the lack of a significant group difference in our study may be attributable to the preoperative selection of the cohort, comprising only cases with characteristics likely to account for the risk of a “non-near complete” vitreous removal.

Regarding our secondary outcomes, a significant improvement in BCVA was seen at the end of follow-up in both groups. The CMT was significantly higher in group B than group A at the m3 visit, and the presence of cystoid macular edema in group B cases necessitated significantly more local steroid injections than in group A. The return to baseline CMT values in both groups at the m6 evaluation indicated that the increase therein was transient, and that the subtenon injection of triamcinolone acetonide was safe and effective particularly when applied to pseudophakic eyes (group B). These results regarding CMT may accord with the postoperative inflammation described in other series of PCV [7, 15, 26]. We did not investigated the possible presence of metamorphopsia or microperimetry alterations, as recently performed by Borowicz et al. in a cohort of vitrectomized RRD [27].

In the literature, the average delay between PPV (for any reason) and subsequent cataract extraction ranges from 16 to 24 months, which may justify preservation of a healthy crystalline lens at the time of PPV in selected cases [6, 28]. However, cataract formation can occur even much earlier, regardless the caliber of the instruments and with a mechanism not completely understood [29]. The protective role of the vitreous gel against the direct interaction between the molecular oxygen from the retinal vasculature and the lens has been considered [30, 31]. In the light of these considerations, the management of a RRD while preserving the lens still involves scleral buckling; the preference with respect to vitrectomy often takes into account the absence of a preexisting PVD [5].

Preservation of the crystalline lens at the time of PPV in our RRD patients ensured similar outcomes to those obtained with PCV, in terms of the retinal reattachment rate and safety during postoperative management. However, the RR data for retinal detachment recurrence indicated that the possible superiority of combined surgery (PCV) should be discussed with the patient preoperatively. At the end of follow-up, the BCVA was similar between the two groups, although recovery over the follow-up was more rapid in group A. Sparing the natural lens was not associated with rapid worsening of the cataract. At the end of follow-up, lens opacity was more advanced than at the baseline, but the change was less than two LOCS III grades on average.

The limitations of this study included the relatively small sample size and short-duration follow-up preventing long-term outcome assessment. This particularly referred to eyes with preserved lens because of their potential for cataract progression and difficulties in performing the phacoemulsification in vitrectomized eyes. Moreover, retinal cauterization technique was not exactly the same in the two groups. Cryopexy was used in the lens-sparing group, while endolaser was performed in the case of PCV. This because, when there was no risk of lens contact, an ab interno extensive encircling of the rhegmatogenous areas was planned.

## Conclusion

To treat phakic rhegmatogenous retinal detachment, mostly in non-presbyopic patients, parsplana vitrectomy (PPV) can be performed with or without removing the lens. In this randomized study comparing PPV with versus without phacoemulsification, the difference in the rate of retinal reattachment after 6 months was not statistically significant (*p* = 0.3). The relative risk for retinal detachment recurrence indicated that the possible superiority of combined surgery (phacovitrectomy) should be discussed with the patient preoperatively.

## Data Availability

The data that support the findings of this study are available from the corresponding author, PM, upon reasonable request.

## Abbreviations

AL: Axial length
ANOVA: Analysis of variance
BCVA: Best-corrected visual acuity
BIOM: Binocular indirect ophthalmomicroscope
CMT: Central macular thickness
IOL: Intraocular lens
IOP: Intraocular pressure
LOCS III: Lens Opacities Classification System III
M: Month
OCT: Optical coherence tomography
PCV: Phacovitrectomy
PFCL: Perfluorocarbon liquid
PPV: Parsplana vitrectomy
PVD: Posterior vitreous detachment
PVR: Proliferative vitreoretinopathy
RR: Relative risk
RRD: Rhegmatogenous retinal detachment
SD: Standard deviation
SRK/T: Sanders–Retzlaff–Kraff/theoretical formula
W: Week
